# Supplementary material for: Counting complete? Finalising the plant inventory of a global biodiversity hotspot
Source: PeerJ. 2017 Feb 21;5:e2984. doi: 10.7717/peerj.2984 (PMC5322757; doi:10.7717/peerj.2984)
Supplement: Table S1 [file peerj-05-2984-s001.docx]

**S1 Table.**

| **Family** | **Genera included** | **Number of species included** |
| --- | --- | --- |
| ASTERACEAE | *Anthanasia, Bryomorphe, Chrysocoma, Disparago, Elytropappus, Felicia, Gymnostephium, Hymenolepis, Mairia, Metalasia, Osmitopsis, Stoebe, Ursinia, Zyrphelis* | 269 |
| BRUNIACEAE | *Audouinia, Berzelia, Brunia, Linconia, Staavia, Thamnea* | 78 |
| BORAGINACEAE | *Lobostemon* | 29 |
| CYPERACEAE | *Ficinia, Tetraria* | 96 |
| FABACEAE | *Aspalathus, Amphithalea, Liparia, Podylyria, Rafnia, Xiphotheca* | 378 |
| GEISSOLOMATACEAE | *Geissoloma* | 1 |
| GRUBBIACEAE | *Grubbia* | 3 |
| IRIDACEAE | *Bobartia, Freesia, Geissorhiza, Hesperantha, Ixia, Klattia, Nivenia, Sparaxis, Thereianthus, Tritonia, Tritoniopsis, Witsenia* | 284 |
| ORCHIDACEAE | *Ceratandra, Evotella, Pterygodium* | 32 |
| PENAEACEAE | *Brachysiphon, Endonema, Glischrocolla, Penaea, Saltera, Sonderothamnus, Stylapterus* | 23 |
| POACEAE | *Ehrharta, Pentameris* | 82 |
| POLYGALACEAE | *Muraltia* | 109 |
| PROTEACEAE | *Aulax, Diastella, Leucadendron, Leucospermum, Mimetes, Orothamnus, Paranomus, Protea, Serruria, Sorocephalus, Spatalla, Vexatorella* | 329 |
| RESTIONACEAE | *Anthochortus, Askidiosperma, Cannomois, Ceratocaryum, Elegia, Hydrophilus, Hypodiscus, Mastersiella, Nevillea, Platycaulos, Restio, Rhodocoma, Soroveta, Staberoha, Thamnochortus, Willdenowia* | 342 |
| ROSACEAE | *Cliffortia* | 125 |
| RUTACEAE | *Acmadenia, Adenandra, Agathosma, Clausena, Coleonema, Diosma, Empleurum, Euchaetis, Macrostylis, Phyllosma, Sheilanthera* | 254 |
