# Supplementary material for: Counting complete? Finalising the plant inventory of a global biodiversity hotspot
Source: PeerJ. 2017 Feb 21;5:e2984. doi: 10.7717/peerj.2984 (PMC5322757; doi:10.7717/peerj.2984)
Supplement: Table S2 — Values of plotted data based on ‘moving average functions’ (sensu Joppa, Roberts & Pimm, 2011a), presented in Figs. 1A–1D which shows trends over time (1750–2015), in species discovery and taxonomic effort in the Cape Floristic Region (South Africa). See main text for details. [file peerj-05-2984-s002.docx]

**S2 Table.**

| **Start Year** | **Species** | **Cumulative Species** | **Taxonomists** | **Species per Taxonomist** |
| --- | --- | --- | --- | --- |
| 1755 | 30 | 40 | 2 | 14 |
| 1760 | 33 | 70 | 3 | 12 |
| 1765 | 40 | 103 | 4 | 10 |
| 1770 | 35 | 143 | 5 | 7 |
| 1775 | 37 | 177 | 6 | 6 |
| 1780 | 38 | 214 | 6 | 6 |
| 1785 | 48 | 252 | 6 | 8 |
| 1790 | 30 | 301 | 7 | 5 |
| 1795 | 52 | 331 | 8 | 6 |
| 1800 | 63 | 383 | 10 | 6 |
| 1805 | 74 | 445 | 11 | 7 |
| 1810 | 43 | 519 | 10 | 4 |
| 1815 | 31 | 563 | 10 | 3 |
| 1820 | 27 | 593 | 11 | 2 |
| 1825 | 44 | 620 | 15 | 3 |
| 1830 | 94 | 664 | 17 | 6 |
| 1835 | 106 | 759 | 14 | 8 |
| 1840 | 96 | 865 | 12 | 8 |
| 1845 | 41 | 961 | 9 | 5 |
| 1850 | 31 | 1002 | 8 | 4 |
| 1855 | 44 | 1033 | 6 | 8 |
| 1860 | 57 | 1077 | 7 | 9 |
| 1865 | 44 | 1134 | 6 | 7 |
| 1870 | 24 | 1177 | 5 | 4 |
| 1875 | 9 | 1201 | 3 | 3 |
| 1880 | 11 | 1210 | 3 | 4 |
| 1885 | 15 | 1221 | 5 | 3 |
| 1890 | 35 | 1236 | 7 | 5 |
| 1895 | 48 | 1271 | 11 | 4 |
| 1900 | 46 | 1318 | 10 | 5 |
| 1905 | 42 | 1365 | 10 | 4 |
| 1910 | 35 | 1407 | 8 | 4 |
| 1915 | 39 | 1442 | 9 | 4 |
| 1920 | 39 | 1481 | 9 | 4 |
| 1925 | 53 | 1520 | 10 | 5 |
| 1930 | 47 | 1573 | 9 | 5 |
| 1935 | 40 | 1620 | 9 | 4 |
| 1940 | 45 | 1660 | 9 | 5 |
| 1945 | 68 | 1705 | 8 | 9 |
| 1950 | 57 | 1773 | 6 | 8 |
| 1955 | 62 | 1830 | 5 | 10 |
| 1960 | 48 | 1892 | 5 | 8 |
| 1965 | 63 | 1940 | 7 | 9 |
| 1970 | 47 | 2003 | 6 | 7 |
| 1975 | 48 | 2050 | 8 | 6 |
| 1980 | 73 | 2098 | 11 | 7 |
| 1985 | 76 | 2170 | 13 | 6 |
| 1990 | 70 | 2247 | 12 | 5 |
| 1995 | 38 | 2316 | 10 | 4 |
| 2000 | 31 | 2354 | 10 | 3 |
| 2005 | 27 | 2385 | 9 | 3 |
